# Foundations And Task Forces

**DOI:** 10.4103/0973-1229.32151

**Published:** 2007

This chapter looks into how Foundations are influenced by Industry in the light of the case of the American Heart Foundation, Genentech and the drug Altepase. It also discusses important ramifications of the reports of two Task Forces of the AAMC, which discuss individual and institutional conflict of interest.

## American Heart Foundation And Genentech

We saw in the last chapter how CPGs are manipulated. But they are not the only area of concern. Even the actions of Foundations that work for disease amelioration have come under some scrutiny. For example disease-specific foundations such as the American Heart Association are heavily funded by industry. This Foundation received US$ 11 million in donations from Genentech, a firm that was very interested in the foundation's acute stroke management guideline, which then went on to recommend the company's product (Baird, 2003; Lenzer, 2002).

Lenzer (2002) makes the following summary points how the Foundation's guidelines were influenced by industry to promote their product Alteplase:

The American Heart Association rated the thrombolytic agent alteplase (tPA) as a class I (definitely recommended) intervention for stroke despite controversy about its safety and efficacy

Most of the association's stroke experts have ties to the manufacturers of alteplase

Genentech, the US manufacturer of alteplase, contributed over $11m to the American Heart Association in the decade before its recommendation on alteplase

Following public scrutiny, the American Heart Association recently withdrew statements that alteplase for stroke “saves lives”

Seemingly impartial organisations that issue professional guidelines may have ties to the manufacturers of recommended interventions

Interesting parallels can be drawn here with the recent case of Aprotinin (Trasylol) and rhAPC (Xigris) we detailed in Chapter I and II respectively.

Studying the panelists for guidelines about the drug, the researchers found out:

A panel of nine was responsible for the guidelines, eight supporting alteplase and one dissenting. Independent investigation for this article shows that six of the eight panellists who supported alteplase for stroke as a class I recommendation had ties to the manufacturer. Four panellists received lecture fees as members of the Genentech speakers bureau; one serves as a consultant to Boehringer Ingelheim, a “development and marketing partner” with Genentech in producing and distributing alteplase; and two received research funding from Genentech (some panellists had more than one form of relationship with Genentech.) Only two of the panellists who supported the upgraded classification had no ties to the manufacturer. Dr Jerome Hoffman, the lone dissenting panellist, also had no industry ties (Lenzer, 2002).

Two-thirds members conflicted. It is interesting to note the last sentence:

Dr. Jerome Hoffman, the lone dissenting panellist, also had no industry ties.

Well, how much more will lucre rule research? How much more will academia, either willingly or unwittingly, give in to unscrupulous elements in industry? While it is invidious to paint the total industry black, it is equally important such cases do not go unnoticed or get connived at. For, that may serve a short-term gain but will be disastrous for long-term growth of both parties.

An interesting example of academia naïveté or cover up, whatever, is below:

 Two panellists initially denied receiving Genentech funding or fees. One, who received lecture fees, acknowledged speaking for Genentech only after being told of evidence of his relationship: “I didn't realise I was officially on the speakers bureau.” When asked the time frame of his lectures, he responded, “Mostly between 1997 and 2000.” Another panellist denied being a principal investigator on a Genentech-sponsored trial, only acknowledging this role after being told that a coauthor had identified him as a principal investigator and after receiving a copy of the original article listing him as such. He said he had enrolled only a few patients, then withdrew from the study and didn't realise his name was listed as a principal investigator in JAMA (Lenzer, 2002).

Well. Well. Well.

## Got The Message?

Got the message, we hope. The guidelines of your foundation promote my product, I will see to it your financial interests are taken care of. You scratch my back, I scratch yours.

Although to be fair to the American Heart Foundation and add to our discomfort, let us note that funding of professional organizations as well as non-profit making health care bodies is quite widespread. The American Cancer Society, for example, ‘is funded by AstraZeneca, Johnson and Johnson, Bristol-Myers Squibb, Eli Lilly and other manufacturers of diagnostic tests and treatments for prostate cancer. Breast Cancer Awareness Month is funded by AstraZeneca, the manufacturer of Nolvadex (tamoxifen), while Eli Lilly, manufacturer of fluoxetine (Prozac) along with 17 other manufacturers of psychoactive drugs, provided $11.72m (£8.3m, €13.4m) to the National Alliance of the Mentally Ill’ (Lenzer, 2002). The case of other such organisations at other places is not likely to be dissimilar. The recent example of the well orchestrated campaign for Xigris by Elli Lilly of a similar nature is detailed elsewhere (See chapter III, p 45-47).

### Welcome Whistle Blowing Papers

We would love to believe these are stray incidents in an otherwise healthy relationship and we would not like to be rabble-rousers or sound undue alarm bells. But these incidents make it important that more research to expose such misdemeanours is undertaken and journals and their editors welcome such papers as legitimate research. For that they may have to muster the courage to displease present and potential sponsors whose business interests get jeopardized by such revelations. Easier said than done. Because, for that, publishers/editors will have to manage journals without succumbing to hidden agendas of sponsors. And for that, docs and the rest will have to pay their way through, not just heightened journal subscriptions, also the rest of the cosy facilities they enjoy. That too raises costs for end-users, the patients and other payers.

So what's the difference between this and the present situation? A legitimate question. The difference is it will be without manipulation by profit seeking sponsors. However, resistance from patients and other payers will have to be met by docs and publishers. Sponsors manage it all because of their strong profit motive and marketing tactics. How do docs and publishers/editors manage it? They have so many other tasks at hand. And they are not marketing experts. So it serves them well to allow the status quo to prevail, where all parties concerned have a ball and occasional alarm bells are suitably sounded. And all welcome them as necessary defuse mechanisms. While the merry band wagon keeps rolling.

Even though this appears the reality at present, it can change. Which will only be if long-term goals guide major players. How? Short-term players would want huge ethical compromises for fund release. The long term maybe expected to play more according to the rules of the game for they have benefited by genuine biomedical advance earlier and would want to enjoy its fruits again. That is why they are still there and medicines still work and biomedical advance is still possible, in spite of so many forces trying to waylay it for personal and pecuniary benefits.

Hence, if the long-term interests of journals and linked biomedical advance are considered, unscrupulous industry players can be shown the door without causing any great dent in financial prospects. For their financial strength rests on their credibility and that is not for sale. Credibility can be marketed, but it can't be bought. Sponsors will be forced to seek such credibility, but without allowing them to compromise it. Like the way a Sachin Tendulkar, the cricketer, plays the game with his sponsors: I am top class. I need the dough which you sponsors can give by marketing me. But I also have values, which are not for sale.

Such is the game journal publishers/editors and genuine researchers will also have to play with sponsors. But they can do so only if they are thoroughly competent and have abiding faith in an uncompromising set of ethical values.

Sometimes, an example teaches more eloquently than reams of paper.

## AAMC Task Force Reports 2001, 2002

The rapidly increasing trend toward influence and control by industry has become a concern for many others too. It is of such importance to the Association of American Medical Colleges that it has issued two new documents which are reports of AAMC Task Forces on Financial Conflict of Interest in Clinical Research. The first one, on how to deal with individual conflicts of interest (Association of American Medical Colleges, 2001) and the second on how to deal with institutional conflicts of interest (Association of American Medical Colleges, 2002), in the conduct of clinical research.

There is a small tale behind the first report, which is worth recounting here. The AAMC Executive Council approved the First Task Force report in December 2001. It was endorsed by 26 of its 28 members. Why not all 28? One member, Hedrick Smith, withdrew because of professional work during the Sept 11 attacks. Understandable. The other did not withdraw but declined to endorse. She was Susan Hellmann, Chief Medical Officer of Genentech. She did so ‘due primarily to her concern that the recommendations present an impediment to research innovation’ (AAMC Financial Conflicts of Interest in Clinical Research, 2007).

We talked of Genentech in relation to the American Heart Foundation earlier in this chapter itself. Need we say more?

What is laudable is the AAMC went ahead and published what it had to. What is equally laudable is the Task Force received a grant, not from industry, but from a medical institute, the Howard Hughes Medical Institute. And so it becomes an important document to peruse.

## The 2001 Report

### Human Subjects, Public Confidence And Competing Interests

In the 2001 document of the task force, the introduction makes it clear that safety and welfare of research subjects is paramount and should never be subordinated to financial interests or personal gain. Also, gaining and retaining public confidence in academia's role in research is equally important:

Institutions in which faculty, staff or students conduct research involving human subjects must ensure that the safety and welfare of those subjects and the integrity of the research are never subordinated to or compromised by, financial interests or the pursuit of personal gain. The AAMC Task Force on Financial Conflicts of Interest in Clinical Research acknowledges significant ongoing public concern about the existence of financial interests in human subjects research and strongly encourages academic institutions to respond in ways that instill confidence in their capacity to identify these interests and to manage them safely and effectively (Association of American Medical Colleges, 2001. p3).

While acknowledging that research generates an inevitable conflict of interest, the task force focuses on the way financial interests can affect patient enrolment, their clinical care, confidentiality of health information, scientific integrity, bias in study design, data collection/analysis, reporting of adverse events and publication of findings:

Competing interests, particularly those engendered by a desire to advance scientific knowledge or to achieve professional recognition, are an inescapable fact of academic life. Most are managed through institutional policies and practices and through the constraints imposed by the scientific method. Yet financial interests in human subjects research are distinct from other interests inherent in academic life that might impart bias or induce improper behavior, because financial interests are discretionary and because the perception is widespread that they may entail special risks. Specifically, opportunities to profit from research may affect - or appear to affect - a researcher's judgements about which subjects to enroll, the clinical care provided to subjects, even the proper use of subjects’ confidential health information. Financial interests also threaten scientific integrity when they foster real or apparent biases in study design, data collection and analysis, adverse event reporting or the presentation and publication of research findings (ibid. p3).

The crucial question is how do institutional policies and practices, as also the constraints placed by the scientific method, both excellent ideas, help when they are subverted, sometimes overtly sometimes subtly, by smart but unscrupulous operators on both sides, in academia as well as industry. For it is naïve to believe only one side is the culprit. Equally naïve is it to believe only appealing to the good sense of people on both sides will work. Some concrete guidelines will have to be laid and more importantly, a statutory regulatory process put into place whose actions are themselves under scrutiny. Of course, this involves the dilemma of infinite regress, but somewhere down the line, methods and policies must follow pious platitudes. Even if there be processes and people waiting to subvert it.

No cynicism, or skepticism, should be allowed to abort effort in this connection. For this is the biggest drawback of the idealist: he knows what is right, but convinces himself it cannot be implemented seeing the atmosphere around. And sinks into moral torpor by cynical portrayal of the behaviour of contemporaries. This must be firmly resisted as an attitude option, as much in self as in others.

### Industry Sponsorship, Academia Self-Interest And Social Good

Coming back to the AAMC Task Force Report, it seems to, at the same time, look favourably at industry sponsorship, since it fills the gap between basic research funding by public funds and the development of diagnostic, therapeutic and preventive products, where industry funding enters into the picture:

At the same time, a principled partnership between industry and academia is essential if we are to preserve medical progress and to continue to improve the health of our citizenry. The generous public support of scientific research in America's universities since World War II has been predicated on the expectation that scientific advancements will yield tangible public benefits - a robust economy, strong national security and a healthy citizenry. Yet, public research support is, for the most part, purposefully limited in scope to basic research and essentially ceases at the point at which scientific invention enters the pathway of product development. In biomedicine, with rare exceptions, it is the private sector, not academia, that develops diagnostic, therapeutic and preventative products and brings them to market. At the crucial interface between innovation and development, researchers from academic medicine often play a critical role by conducting the early translational research that gives rise to new products and by testing these novel products for safety and efficacy (ibid. p3-4).

While acknowledging that biotechnology and information technology have both raised expectations in the public mind from research institutions, they have also raised hopes that academia will be unaffected by financial self-interests and serve the social good alone, which expectation is likely to be belied as industry influences researchers and research subjects:

The growth of the biotechnology industry is a celebrated accomplishment of the U.S. economy during the second half of the 20th century and together with the information technology industry has spurred public perception of research universities as engines of economic development and social betterment. But at the same time, the public insists that universities remain unblemished by financial self-interest and continue to serve society as trusted and impartial arbiters of knowledge. This “conflict of public expectations” is nowhere more intense than in academic medicine and in research involving human subjects, where the steadily deepening engagement of clinical research with the world of commerce is seen by many influential observers as threatening both research integrity and the welfare of research participants (ibid, p22).

The Task Force does not eschew academia-industry connect, in fact it expects it to deepen in a number of important areas, but the need to maintain public confidence and trust are critically important if this connect is to prosper:

The Task Force acknowledges the enormous benefits that have inured to the public from the commercial development of medical inventions made in academic medical centers and anticipates that the relationships of these centers with industry will only continue to deepen in an era in which terms like genomics, proteomics and physiomics are becoming commonplace. But the Task Force also recognizes that the public's extraordinary support of academic biomedical research will remain critically dependent upon public confidence and trust that are especially vulnerable in research involving human subjects. This is the reality and it must be appreciated by industry as much as by academe if their future interactions are to thrive (ibid, p22).

### To Summarise The 2001 Report

To summarise the salient points of the 2001 Task Force relevant to our discussion:

1. Safety and welfare of research subjects is paramount and should never be subordinated to financial interests or personal welfare;
2. While acknowledging that research generates an inevitable conflict of interest, gaining and retaining public confidence in academia's role in research can affect patient enrolment, their clinical care, confidentiality of health information, scientific integrity, bias in study design, data collection/analysis, reporting of adverse events and publication of findings;
3. lndustry sponsorship is necesssary, since it fills the gap between basic research funding by public funds and the development of diagnostic, therapeutic and preventive products, where industry funding enters into the picture;
4. Biotechnology and information technology have raised expectations in the public mind from research institutions, but they have also raised hopes that academia will be unaffected by financial self-interests and serve the social good alone;
5. The Task Force does not eschew academia-industry connect, in fact expects it to deepen in a number of important areas; but the need to maintain public confidence and trust are critically important if this connect is to prosper.

## The 2002 Report

### Serve as Public Trust, Reconcile Competing Interests, Avoid Bias

In the 2002 document of the AAMC Task Force, the concerns that academia should voice are spelt out - serving as a public trust while carrying out their diverse obligations; and, avoiding conflict of competing interests or getting biased or tainted:

Academic institutions are privileged to serve as a public trust for the advancement, preservation and dissemination of knowledge. These institutions have diverse obligations: to students, faculty and staff; to legislators and regulators; to donors and benefactors; and to society at large. When meeting these obligations in the ordinary course of business, institutions must and do reconcile competing interests. In so doing, institutions recognize widely that policies must be made and decisions taken in a manner that is free of the taint of improper bias or conflict of interest (Association of American Medical Colleges, 2002. p1).

### ‘Institutional’ Conflict of Interest

The concerns voiced by patient advocates and their votaries, over the greater financial dealings between academia and research sponsors has given rise to ‘institutional’ conflict of interest which pose risk to research subjects and research integrity and which present institutional processes may be incapable of handling:

Increasingly, academic institutions that conduct research also invest in - and accept the philanthropy of - commercial research sponsors. Regulators, legislators, journalists and patient advocates have now begun to question whether such financial relationships may give rise to “institutional” conflicts of interest that could threaten research integrity and, especially troubling, potentially pose risks to human research subjects. Concern has arisen that existing institutional processes for resolving competing interests may be insufficient when the institution has a financial interest in the outcome of research and the safety and welfare of human subjects are at stake (ibid, p1).

Public support for research may weaken as institutions are perceived to have financial conflicts of interests and it may increase legislative and governmental scrutiny into institutional activities, which they may find rather disconcerting:

Although perceived risks to human subjects have received the greatest attention thus far, the growing perception that research institutions may have financial conflicts of interest also threatens to weaken public support for research. In an era of tremendous public investment in academic research, legislators and policymakers and others justifiably expect heightened public accountability from research institutions (ibid, p1).

Again, concerned that the objectivity of research may get, or appear to get, compromised because of ‘institutional’ financial conflict of interest, the Task Force offers principles to balance fiduciary and ethical responsibilities related to research on human subjects:

The AAMC's Task Force on Financial Conflicts of Interest in Clinical Research believes that an institution holding certain financial interests related to its human subjects research may face a conflict of interest when that institution employs the individuals who review, supervise and conduct the research. This is especially troubling because the regulation of federally sponsored university research is centered on the principles of institutional integrity in the conduct of that research and responsibility and accountability for its oversight. Because the safety and welfare of research subjects and the objectivity of the research could be - or could appear to be - compromised whenever an institution holds a significant financial interest that could be affected by the outcome, the Task Force offers the principles and the recommended processes described in this report as a means to address an institution's competing fiduciary responsibilities and ethical obligations in the context of human subjects research (ibid, p1-2).

Duty to protect human subjects, to ensure integrity of research, compliance with applicable laws and regulations must combine with the institution's financial health and economic viability of academic/research activities. To take care of the inevitable conflicts of interest so generated, institutions have to take care that human subjects and research integrity get precedence over institutional interests:

Institutional policies should affirm that the welfare of human subjects and the integrity of research will not be compromised - or appear to be compromised - by competing institutional interests or obligations (ibid, p3).

To ensure this occurs, institutions should follow a fundamental principle: separate human subject research from investment and licensing decisions:

As a fundamental principle, institutions should ensure that in practice, the functions and administrative responsibilities related to human subjects research are separate from those related to investment management and technology licensing (ibid, p3).

### To Summarise The 2002 Report

To summarise the salient points of the 2002 Task Force relevant to our discussion:

1. Academic institutions must serve as a public trust while carrying out their diverse obligations and all activities undertaken free of conflict of interest or getting biased or tainted;
2. The concerns, voiced by patient advocates and their votaries, over the greater financial dealings between academia and research sponsors, has given rise to ‘institutional’ conflict of interest, which pose risk to research subjects, and which present institutional processes may be incapable of handling;
3. Public support for research may weaken as institutions are perceived to have financial conflicts of interests and it may increase disconcerting legislative and governmental scrutiny into institutional activities;
4. Concerned that the objectivity of research may get, or appear to get, compromised because of ‘institutional’ financial conflict of interest, the Task Force offers principles to balance fiduciary and ethical responsibilities related to research on human subjects;
5. Duty to protect human subjects, to ensure integrity of research, compliance with applicable laws and regulations must combine with the institution's financial health and economic viability of academic/research activities. To take care of the inevitable conflicts of interest so generated, institutions have to take care that human subjects and research integrity get precedence over institutional interests;
6. Institutions should follow a fundamental principle: separate human subject research from investment and licensing decisions.

## Concluding Remarks

1. A number of disease-specific foundations are heavily funded by industry that creates serious conflict of interest likely to result in favourably recommending therapies of sponsors. The example of Alteplase and the American Heart Foundation is a recent one.
2. Whistle blowing papers and research to expose misdemeanours must be undertaken and journals and their editors should welcome such papers as legitimate research, but without encouraging a witch-hunt.
3. The USP of journals and researchers is credibility. Credibility can be marketed, but it cannot be bought. Sponsors will be forced to seek such credibility without allowing them to compromise it. Such is the game journal publishers/editors and genuine researchers will have to play with sponsors. But they can do so only if they are thoroughly competent and have abiding faith in an uncompromising set of ethical values.
4. The Task Force of the AAMC in its two reports of 2001 and 2002 recognises the necessity and inevitability of the academia-industry connect, but wants to avoid its undesirable influence on the integrity of research and the welfare of human research subjects. It is specially concerned that public, activist and governmental control and concern not cast a spanner in the works of a potentially promising relationship.
